# Drug-target interaction prediction using semi-bipartite graph model and deep learning

**DOI:** 10.1186/s12859-020-3518-6

**Published:** 2020-07-06

**Authors:** Hafez Eslami Manoochehri, Mehrdad Nourani

**Affiliations:** Department of Electrical and Computer Engineering, The University of Texas at Dallas, 800 W Campbell Rd, Richardson, TX, 75080 USA

**Keywords:** Drug-target interaction, Link prediction, Deep learning, Weisfeiler-Lehman algorithm

## Abstract

**Background:**

Identifying drug-target interaction is a key element in drug discovery. In silico prediction of drug-target interaction can speed up the process of identifying unknown interactions between drugs and target proteins. In recent studies, handcrafted features, similarity metrics and machine learning methods have been proposed for predicting drug-target interactions. However, these methods cannot fully learn the underlying relations between drugs and targets. In this paper, we propose anew framework for drug-target interaction prediction that learns latent features from drug-target interaction network.

**Results:**

We present a framework to utilize the network topology and identify interacting and non-interacting drug-target pairs. We model the problem as a semi-bipartite graph in which we are able to use drug-drug and protein-protein similarity in a drug-protein network. We have then used a graph labeling method for vertex ordering in our graph embedding process. Finally, we employed deep neural network to learn the complex pattern of interacting pairs from embedded graphs. We show our approach is able to learn sophisticated drug-target topological features and outperforms other state-of-the-art approaches.

**Conclusions:**

The proposed learning model on semi-bipartite graph model, can integrate drug-drug and protein-protein similarities which are semantically different than drug-protein information in a drug-target interaction network. We show our model can determine interaction likelihood for each drug-target pair and outperform other heuristics.

## Background

Prediction of Drug-Target Interactions (DTI) is a critical part of drug discovery in pharmaceutical research. Compared to biochemical experimental methods which are laborious, time consuming and extremely expensive, computational methods are of high interest because they can efficiently identify potential DTIs or narrow down the search space for biologists and biochemists.

Most of traditional approaches for predicting DTI, either for drug discovery or repositioning (reusing already available drugs for new targets) are ligand-based approaches. These techniques predict drug-target interactions based on the similarity between the target proteins’ ligands [1, 2]. Docking-based methods utilize 3D structure information of a target protein. Ligand’s and docking methods then run simulations to estimate the likelihood that it will interact with a certain drug based on their binding affinity and strength [3, 4]. However, these approaches often lead to poor prediction results when a target has only a small number of known binding ligands. On the other hand, the performance of docking-based approaches is limited to availability of 3D structures of target proteins and can be quite poor.

Machine learning methods for computational prediction of DTI have become more popular in recent years [5, 6]. In these approaches, DTI has been modeled using different techniques such as recommendation systems [7, 8], supervised classification problem [9], bipartite graph [10, 11] and network-based approaches [12, 13].

In recent years, several approaches tried to take advantage of drug chemical structure and protein sequence by integrating them into the known drug-target network in the form of drug-drug and protein similarities. These methods are based on *guilt by association* assumption where similar drugs may share similar targets and vice versa. Mostly, these approaches treated similarity information as input features and formulated the DTI prediction as a binary classification task in which presence of an interaction between drugs and targets is captured. For instance, bipartite local model (BLM) is proposed to model DTI network and a support vector machine is used for prediction task [10]. This work is further extended by Mei et al. by combining BLM with a neighbor-based interaction-profile inferring (NII) technique (called BLMNII) [14]. This method is able to learn the DTI features from neighbors and predict interactions for new drug or target candidates. In another study, Xia et al. proposed NetLapRLS which is a semi-supervised learning method for DTI prediction [15]. NetLapRLS applies Laplacian regularized least square and incorporates both similarity and interaction kernels into the prediction framework. Van Laarhoven et al. introduced a Gaussian interaction profile (GIP) kernel-based approach coupled with RLS for DTI prediction [16, 17]. Zheng et al. proposed a collaborative matrix factorization (MSCMF) for DTI [18]. They incorporated drug and protein similarity matrices to regulate the DTI network. In [19] and [20], random walk with restart algorithm is presented to predict new drug target interactions using known DTI as well as drug-drug and protein-protein similarities and interactions. Network-based Inference (NBI) models the prediction problem as a network where the drugs and targets are represented as nodes, and the interacting drug-target pairs and similarities are represented as edges. The network diffusion technique is then applied to propagate interaction information throughout the drug-target interaction network [21].

A large number of network-based methods, mostly identify DTI based on specific heuristics. For example, BLM uses common neighbors as heuristic by measuring the weighted nearest neighbor. In another study the shortest path between drugs and target is proposed as a heuristic [22]. Recently, Yu et. al [11] investigated the predictive power of similarity indices such as common neighbors and Jaccard Index on predicting DTI, purely based on known DTI information. Although these heuristic make sense in drug-target interaction, they cannot fully reveal the underlying relations between drugs and targets. Very recently, deep learning techniques have gained much attention for their promising performance to learn complex networks such as social and biological networks [23–25]. DTI network is no exception and recently some deep learning based methods are proposed to deal with limitation of handcrafted feature, and similarity metrics [26–28].

Inspired by link prediction methods for complex graphs, in this paper we propose a supervised learning heuristic for drug-target interaction prediction that unlike traditional methods that rely on hand-engineered graph features, it learns the network topology by itself. First, we construct a semi-bipartite graph by exploiting known DTIs and drug-drug and protein-protein similarities. Then, in pre-processing step, we provide positive samples among known interactions and likely negative samples among unknown data. We then propose a sub-graph extraction algorithm to extract sub-graphs for each drug-target pair sample. Our algorithm captures the closest neighbors by considering geometric distances in drug target nodes as well as drug-drug and protein-protein similarities. Each sub-graph represents the graph topology surrounding of each drug-target pair. To learn a meaningful model and preserve the ordering of graph vertices, an ordering mechanism is required to assign similar indices to nodes with similar structural role from different sub-graphs. For this purpose, we employed a graph labeling method to measure the similarity between nodes and sub-graphs. After ordering the vertices, sub-graphs are encoded into embedding vectors. Finally, we use deep neural network to learn nonlinear topological features and complex patterns from the enclosing sub-graphs.

## Methods

### DTI problem formulation

**Fig. 1:**
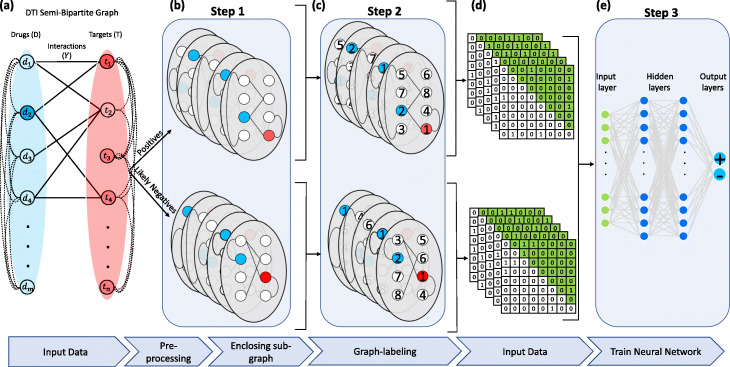
Our model workflow. **a** Data representation. A semi-bipartite graph is constructed by Drug-target interactions, drug-drug similarity and protein-protein similarities. **b** Drug-target positive and negative pairs (extracted based on [29]) samples are represented as sub-graphs capturing the topological environment around drug target pairs. **c** A graph labelling method is applied on each sub-graph in order to preserve the ordering of graph vertices. **d** The final sub-graphs are converted to adjacency matrices and the upper triangle of each matrix is representing embedded features to train a classifier. **e** A deep neural network is trained and used for predicting new drug target pairs

Predicting drug-target interaction can be formulated as link prediction of a bipartite graph in which nodes represent drugs and targets in two sets and the edges denote the interactions. To capture the drug-drug and target-target similarities, we formulate the DTI network as an un-directed semi-bipartite graph *G*=<*D*,*T*,*E*,*F*,*H*>, where *D* and *T* are set of drug (chemical compound) and target (protein) nodes respectively, *E*⊂*D*×*T* is the set of edges (observed links) between *D* and *T*, i.e. *E*={(*d*_*i*_,*t*_*j*_)|*d*_*i*_∈*D*,*t*_*j*_∈*T*}, *F*⊂*D*×*D* is the set of edges between the nodes in *D*, i.e. *F*={(*d*_*i*_,*d*_*j*_)|*d*_*i*_,*d*_*j*_∈*D*} and *H*⊂*T*×*T* is the set of edges between the nodes in *T*, i.e. *H*={(*t*_*i*_,*t*_*j*_)|*t*_*i*_,*t*_*j*_∈*T*}. An example of such a network is shown in Fig. 1a where drug-drug and target-target similarities are integrated into the graph. The drug-target interaction network can be represented by a *m*×*n* adjacency matrix *Y* as follows:

1 $$  y_{ij}=\left\{\begin{array}{ll} 1, & \text{if there is a known \((d_{i},t_{j})\) interaction}\\ 0, & \text{otherwise}. \end{array}\right.  $$

where *y*_*ij*_ denotes the <*i*,*j*>th element of matrix *Y* (1≤*i*≤*m*,1≤*j*≤*n*) and (*d*_*i*_,*t*_*j*_) denotes drug *d*_*i*_ and target *t*_*j*_ pair. The goal here is to assign a score to each *y*_*ij*_ that ultimately help to classify it as whether they interact or not. Note that elements with *y*_*ij*_=1 and *y*_*ij*_=0 correspond to positive and unknown interactions, respectively. Throughout this paper, the set of protein targets that interact with drug *d*_*i*_ and drugs that interact with protein *t*_*j*_ are shown by $T_{d_{i}} \subset T$ and $D_{t_{i}} \subset D$, respectively. Drug-drug and protein-protein similarities, are also represented by *S*^*D*^∈[0,1]^*m*×*m*^ and *S*^*T*^∈[0,1]^*n*×*n*^ matrices, respectively.

### Workflow

Figure 1 presents the proposed framework in this work. After data preparation and constructing the semi-bipartite graph, positive samples are determined randomly from the graph. Negative samples, however, are determined by a method to be discussed in pre-processing step (subsection “Pre-processing”) which selects reliable negatives among unknowns. Then, our learning model is applied to learn drug target interaction from prepared samples. Our method consists of three steps shown in Fig. 1.

1. Extracting enclosing sub-graphs: In this step, for each (*d*_*i*_,*t*_*j*_) pair sample, an enclosing sub-graph with *K* vertices are created to capture the neighboring information of (*d*_*i*_,*t*_*j*_).
2. Encoding sub-graphs: In this step, a vertex ordering is applied on each sub-graph and then the new sub-graphs are converted to embedding vectors.
3. Learning phase: A deep neural network is trained to learn non-linear graph topological features to predict unknown links.

### Pre-processing

One of the challenges to train a model using DTI network is that, only a small number of interactions (positive samples) are known. Those that do not interact with each other are not known (i.e. missing edges in the network). Therefore, in most approaches (e.g. [28, 30–32]), negative samples are chosen randomly from the dataset. However, this might result in inaccurate findings and impact the classifier’s decision boundary. In fact, a study by Liu et. al. [29] showed properly choosing reliable negative samples can drastically improves the performance. This is the case in some approaches such as Bayesian Matrix Factorization [33], BLM [10] and Gaussian kernel profile [17]. In this work, similar to [29], first we identify reliable negative samples. The main idea is the drugs that are dissimilar to every known drug of a given target are not much likely to interact by the target and vice versa. First, we create a pool of negative candidate pairs of drugs and targets. This set excludes the set of known interacting pairs (i.e. corresponding *y*_*ij*_=1). Any negative candidate interaction is defined by a triplet (*d*_*i*_,*t*_*j*_,*s*_*ij*_) where *s*_*ij*_ is a score between drug *d*_*i*_ and target *t*_*j*_. We compute $s^{DT}_{ij} = \sum _{t_{k} \in T_{d_{i}}} S_{t_{j}t_{k}}^{T}$, that sums up similarity of every target that interacts with *d*_*i*_ with *t*_*j*_. Similarly, we compute $s^{TD}_{ji} = \sum _{d_{k} \in D_{t_{j}}} S_{d_{i}d_{k}}^{D}$, that sums up similarity of every drugs that interact with *t*_*j*_ with *d*_*i*_. Finally, a similarity score between *d*_*i*_ and *t*_*j*_ is computed by:

2 $$  s_{ij} = e^{-\left(s^{DT}_{ij}+ s^{TD}_{ji}\right)}  $$

The negative candidate pool is then ranked based on the similarity score computed above in decreasing order and those with the highest values of the score are considered to be the reliable negatives. Using these reliable negative samples and randomly drawn positive samples from known interactions, we will train a neural network classifier.

### Extracting enclosing sub-graph

For each (*d*_*i*_,*t*_*j*_) pair chosen from the graph *G*=(*D*,*T*,*E*,*F*,*H*) where *d*_*i*_∈*D* and *t*_*j*_∈*T*, an enclosing sub-graph $G_{d_{i}t_{j}}$ which is also a semi-bipartite graph is extracted that captures the surrounding environment of (*d*_*i*_,*t*_*j*_). Here, we only consider *E* edges to find neighbors of any drug are target nodes and vice versa. The challenge is how to identify a sub-graph with *K* number of vertices for a drug-target pair considering both DTI and similarity information which are semantically different. *K* is a predefined parameter also called sub-graph size. The most important information are first-order (first-hop) drug-target interaction links from (*d*_*i*_,*t*_*j*_). In the first step, target neighbors of *d*_*i*_, *N*(*d*_*i*_)⊂*T* and drug neighbors of *t*_*j*_, *N*(*t*_*j*_)⊂*D* are added into sub-graph. If the number of vertices in the sub-graph is less than *K*, we construct a pool of vertices (*χ*), consisting of neighbors of nodes that have been included into the sub-graph but their neighbors have not been included yet and will be processed. Then, we sort the pool based on similarity of drugs with *d*_*i*_ and target proteins with *t*_*j*_ (using *S*^*D*^ and *S*^*T*^, respectively) in decreasing order and keep adding to the sub-graph from top of the pool till size of the sub-graph meets *K*. If the number of vertices in the sub-graph is more than *K*, first use graph labeling to impose an ordering for sub-graph, and then reorder it using this order. After that, if $|G_{d_{i},t_{j}}| > K$, the bottom $|G_{d_{i},t_{j}}| - K$ vertices are discarded. At the end, the sub-graph induces by identified vertices. This process is summarized in Algorithm 1.

### Sub-graph pattern encoding


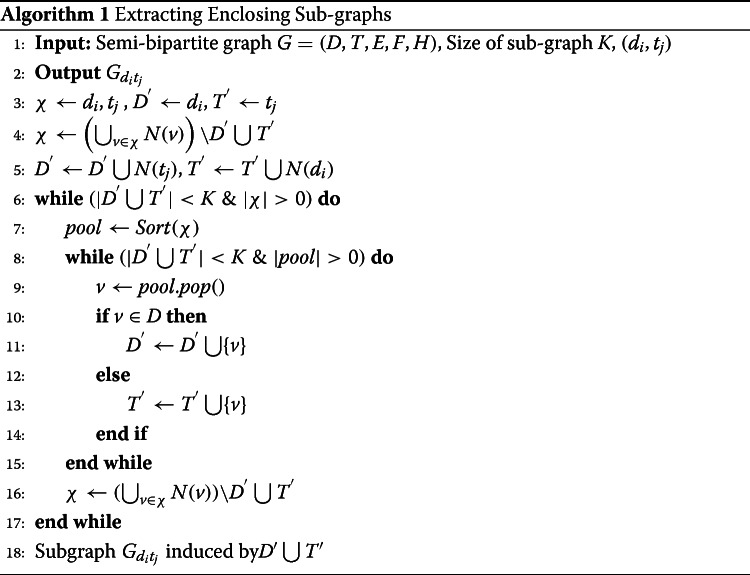


Unlike some recent approaches that provide embedding features for each node of the graph [34], we provide an embedding feature only for each sub-graph representing a drug-target pair’s topological structure. To learn a meaningful model, it is necessary to find a vertex ordering for each sub-graphs. For this purpose, we use graph labeling. The idea is to make vertices from different sub-graphs that have similar structural role, get assigned to similar orders (rankings). A graph labeling function is a map *f*:*V*→*C* from vertices *V* to an ordered set *C*, conventionally called colors in literature. In our problem, *f* must be a one-to-one function, so each vertex is mapped to a unique color.

Among graph labeling algorithms, Weisfeiler-Lehman (WL) algorithm [35] is well-known because of its graph isomorphism test. WL provides vertex ordering based on topological structure of a graph. In this algorithm, initially, all vertices get the same label. Then, in an iterative fashion, each vertex gets a signature string by concatenating its own labels and their immediate neighbors’ labels. Then, signature strings are sorted lexicographically in ascending order and each vertex gets a new label based on its signature string order. For instance, let vertex *x* with label 2 has neighbors with labels {1,2,3} and vertex *y* with label 3 has neighbors with labels {2,2,4}. The signature string of *x* and *y* are {2,123} and {3,224}, respectively. Since, {2,123} is lexicographically smaller than {3,224}, *x* gets smaller label than *y*. This process is repeated until vertices get unique labels. At the end, vertices with similar structural roles get similar labels [36].

Since WL ranks vertices based on topological structure of the graph and structural role of the vertices, it is suitable for any classifier model. WL treats any vertex in the graph identically. However, in our application, we construct each sub-graph for a particular drug-target pair and therefore WL is not able to capture that information. In addition, as WL requires reading and sorting of the vertices’ signature strings, it becomes computationally expensive since the signature strings can be very long for nodes with high degrees. Fast hashing-based WL algorithms were proposed [25, 37] which map unique signature strings to unique real values. To deal with issues mentioned above, we borrowed the Pallete-WL algorithm [25] in which it can take advantage of vertex ordering capability of WL while capturing the core information of each sub-graph (i.e. initial drug-target pair) using a hashing function.

**Fig. 2:**
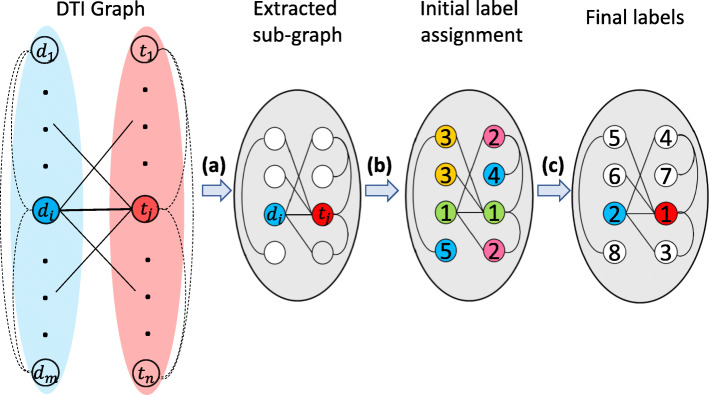
An example of our approach. **a** Extracting sub-graph from the semi-bipartite graph (Algorithm 1). **b** Assign initial colors to vertices according to their geometric mean distance to the link. **c** Refine the colors to impose a vertex ordering which preserves the initial color order

In Pallete-WL, initially, geometric mean distance of any node in the sub-graph $G_{d_{i}t_{j}}$ to *d*_*i*_ and *t*_*j*_ is computed. Then, distance values are mapped to colors by function *f*. Function *f* first maps the smallest real number to color 1, and then maps the second smallest real number to color 2, and so on until every real number is mapped to a color. If two or more real numbers are equal, they are mapped to the same color. Then, a refinement process is iteratively done by mixing their original colors and nearby colors in such a way that the colors’ relative ordering is preserved. This process is driven using a hash function [25]. An example of this algorithm is shown in Fig. 2. In this example, first a sub-graph is extracted for (*d*_*i*_,*t*_*j*_) pair from the semi-bipartite graph. Then, labels for vertices in the sub-graph are assigned based on their geometric distances to *d*_*i*_ and *t*_*j*_. Finally, by the refinement process, each vertex is assigned to a unique label.

After vertex ordering is done on sub-graphs with *K* vertices, sub-graphs are encoded to adjacency matrices with size of *K*×*K*. Each matrix includes {0,1} for (*d*_*i*_,*t*_*j*_) indices, depending of the existence of an edge between them, and values in (0,1] range for (*d*_*i*_,*d*_*k*_) and (*t*_*j*_,*t*_*k*_) indices (using *S*^*D*^ and *S*^*T*^). As the matrices are symmetric, only upper-triangle part is used (Fig. 1d) and vertically converted to $\frac {K(K-1)}{2}$ vectors.

### Learning phase by neural network

After we encode the enclosing sub-graphs and identify embedding vectors for positive (*d*_*i*_,*t*_*j*_) pair samples ((*d*_*i*_,*t*_*j*_)∈*E*) and negatives (*d*_*i*_,*t*_*j*_) pair samples (when (*d*_*i*_,*t*_*j*_)∉*E*), we feed the information into a deep neural network to learn the non-linear topological patterns. After the training phase, interaction for any given drug-target pair can be predicted by the trained neural network. The output of neural network would give us a probability estimate to predict the interaction between testing drug-target pair (i.e. positive or negative - see Fig. 1e).

### Datasets

We adopted a well-known dataset for prediction and evaluation of our DTI prediction method. This dataset has been constructed by [32]. This dataset includes drug-protein interaction network (extracted from the DrugBank database Version 3.0 [38]). It also includes drug chemical structure similarity network (i.e. a pair-wise chemical structure similarity network measured by the dice similarities of the Morgan fingerprints with radius 2, which were computed by RDKit [39]), and protein sequence similarity network (which was obtained based on the pair-wise Smith-Waterman scores [40]). DTI network consists of binary edge weights (i.e. 1 represents a known interaction, and 0 otherwise) and the drug structure similarity network and the protein sequence similarity network consist of real-valued edge weights between 0 and 1. This datasets include 708 drugs, 1,512 protein targets and 1,923 known drug-target interactions. These datasets have widely been used by researchers [28, 41, 42].

## Results

### Performance evaluation metrics and protocols

We used a neural network architecture with three fully-connected layers with 32, 32 and 16 hidden neurons, respectively. For neurons’ activation, we used Rectified Linear Unit (ReLU). A softmax layer is used as the output layer (i.e. assigns estimated probability to each class). These hyper parameters are selected empirically based on trial and error.

After training the neural network, we can predict the interaction between any testing drug-target pair. Similar to training phase, first, we extract enclosing sub-graph for testing pairs. Then, we use our encoding methodology to construct the feature embedding sub-graphs and feed them to the neural network. Neural network provides a prediction score for (*d*_*i*_,*t*_*j*_), which represents the estimated likelihood of interaction. In our paper, for all experiments, 10-fold cross validation is used to estimate the performance of our method on the data. In this method, the data is divided into 10 non-overlapping subsets. 9 out of these 10 subsets are used for training and the remaining 1 subset is used for testing. Positive samples are randomly selected from known drug-target interactions and negative samples are selected based on the method explained in Subsection “Pre-processing”. Like other researchers in this field, we employed the Area Under Receiver Operating Characteristic (AUROC) curve and Area Under Precision-Recall (AUPR) curve to evaluate prediction performance for all methods [43]. In general, ROC curves show the trade-off between the true positive rate (TPR) and false positive rate (FPR), and PR curves show the trade-off between the precision and recall using different probability thresholds.

**Fig. 3:**
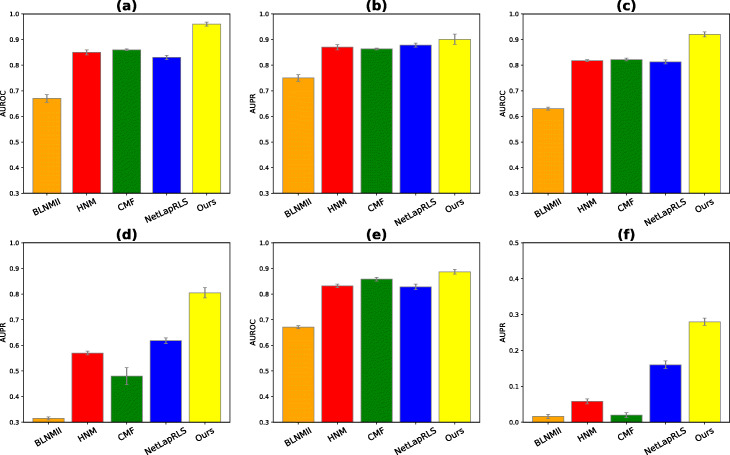
10-fold cross-validation performance evaluation of our approach compared with baseline methods in terms of AUROC and AUPR. **a** AUROC and **b** AUPR scores, in which all methods are trained and tested on balanced datasets. **c** AUROC and **d** AUPR scores in which number of negative samples was 10 times more than the number positive samples (*α*=10*%*). **e** AUROC and **f** AUPR scores in which all unknown drug-target interacting pairs are considered (*α*=0.18*%*). All results were summarized over 10 trials and expressed as mean ± SD

We comprehensively compared our approach with four baseline methods in drug-target interaction predictions reported in literature, namely BLMNII [14], CMF [18], HNM [44] and NetLapRLS [15]. First, we compared the performance of our method with others when the data is balanced (i.e. number of positive and negatives are roughly equal). The AUROC and AUPR results show our approach achieved higher performance than other methods (Fig. 3a-b).

In practice, DTI network is often very sparse with only few known DTIs. To mimic this imbalanced data situation, we randomly sample negative pairs 10 times more than positive pair samples [28] (positive to negative ratio *α*=10*%*). As expected, in all methods, both AUROC and AUPR scores decreased in compared to the case that number of positives and negatives were balanced (Fig. 3c-d). Although in our method AUROC and AUPR scores dropped around 4% and 10% respectively, we observed our method still outperformed other methods with significant improvement.

To further mimic the practical situation and decrease the positive to negative ratio, we chose all unknown interactions as negative samples. In this case, the positive to negative ratio *α*≃0.18*%*. The performance of this setup is shown in Fig. 3e-f. We observed that in this case, our method achieved a higher performance over baseline methods as well. As stated in [17, 32], in this case that the dataset is highly unbalanced, AUPR can provide a better assesment than AUROC metric. The reason is in this scenario, there are many more negatives than positives and AUPR does not account for true negatives. Although the performance of most methods in terms of AUROC are comparable (Fig. 3e), our approach significantly achieved better performance in terms of AUPR (Fig. 3f).

**Fig. 4:**
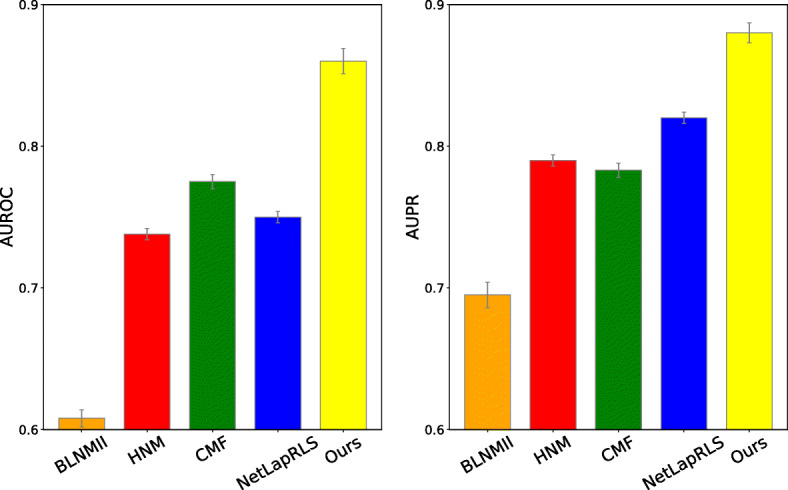
Performance evaluation of our model in terms of AUROC and AUPR in the case that drug-target interactions with similar drugs and proteins are removed

Since the datasets may contain redundant DTIs (i.e. a same protein is connected to more than one similar drugs and vice versa), the performance of prediction can be inflated. To analyze the robustness of our algorithm against removal of homologous proteins or similar drugs, we performed an experiment similar to [28] and [32], in which DTIs with similar drugs (i.e. drug structural similarity) >60% or similar proteins (i.e. protein sequence similarity) >40% are removed. The removal operations reduced the number of interactions from 1,923 to 900. Similar to other experiments, 10-fold cross validation is used to provide AUROC and AUPR performance (shown in Fig. 4). The results indicates our approach outperformed other prediction methods in term of both AUROC and AUPR. As expected, compared to non-removal case, prediction performance is decreased (Fig. 3a-b).

**Fig. 5:**
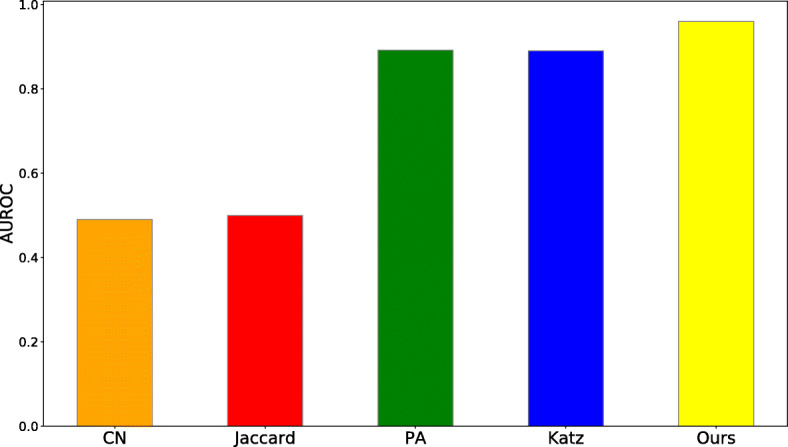
Comparing AUROC performance of our method with other heuristic-based methods

As our model lies under the category of heuristic based approaches, we further compared the performance of our model with other heuristics employed in DTI prediction by Lu et. al [11]. These heuristics used for link prediction which can be categorized into first-order, second-order and high-order heuristic methods, based on the most distant node necessary for computing the heuristic [32]. Namely, heuristics proposed for DTI prediction in [11] are Preferential Attachment (PA) (i.e. first-order heuristic) [45], modified common neighbors (CN) and modified Jaccard Index (i.e. second-order heuristic) and Katz Index (i.e higher-order heuristic). The results illustrated in Fig. 5 show our model outperforms other heuristics in terms of AUROC (as AUPR performance for all other methods were close to zero, this metric is not shown). This is expected as [24] shows, learning high-order heuristics is feasible with a small sub-graph size (*K*) using WL algorithm.

**Fig. 6:**
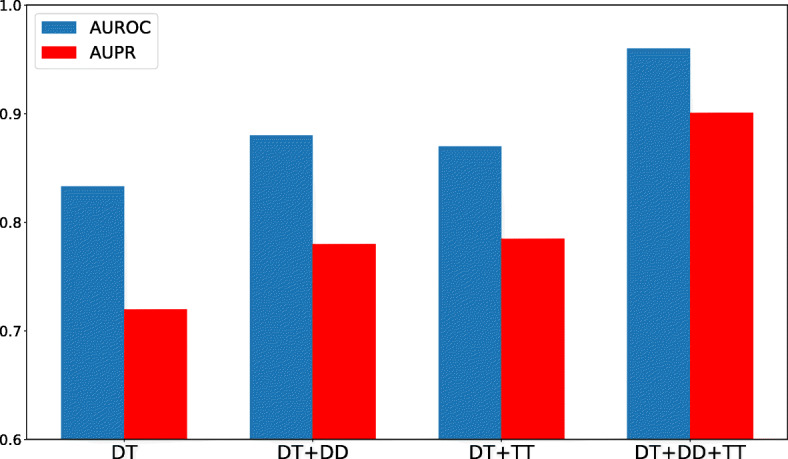
Incorporating the drug-drug structure similarity network (DD) or/and protein sequence similarity network (TT) can improve the prediction performance of our approach

To show the effect of similarity information in our model, we conducted an experiment based on only drug-target (DT) interaction network (i.e bipartite-graph), DT interaction network with drug-drug structural similarities (DD), DT interaction network with protein sequence similarities (TT) and all networks. The results are shown in Fig. 6. It shows additional networks such as drug or/and protein (target) similarity matrices improve the prediction performance. We observed 14% and 18% improvement when all networks are used compared to when only DT network is used in terms of AUROC and AUPR, respectively. Also, this experiment evaluates the robustness of our approach by providing different types of networks.

**Fig. 7:**
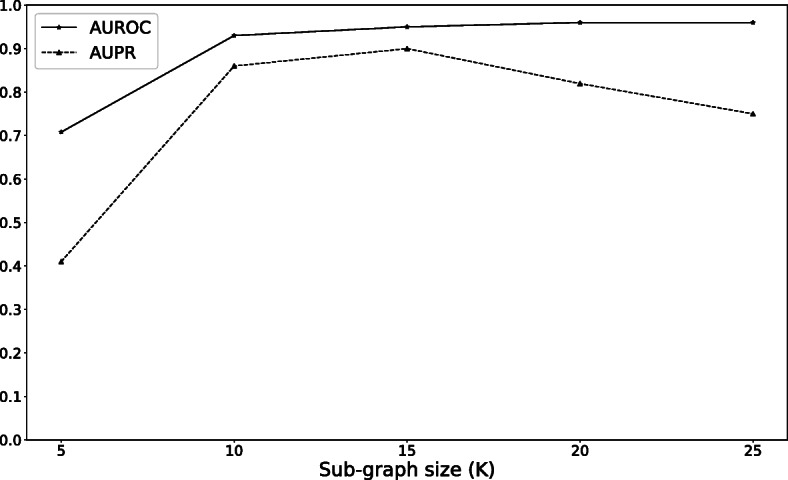
The effect of number of vertices in sub-graph on prediction performance

As our proposed model relies on topological features, we investigated the effect of the size of sub-graph representing drug-target pair in prediction task. Figure 7 shows the overall trend that as the number of vertices in sub-graphs increases, the AUROC performance also increases. However, the performance of our model for *K*>15 remains flat. It is also observed that AUPR score decreases for *K*>15. The trend shown in our work confirms a study by Zhang et. al [24] that shows the most useful information is provided by closer vertices to the link being predicted by WL algorithm. Specifically, we see a diminishing return for AUPR for large values of *K* due to overfitting.

**Fig. 8:**
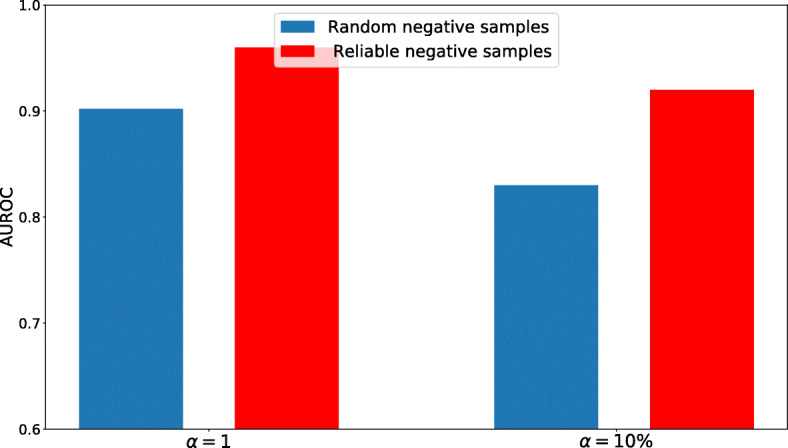
Comparison of the performance of our model with random negative samples and reliable negative samples in terms of AUROC

**Fig. 9:**
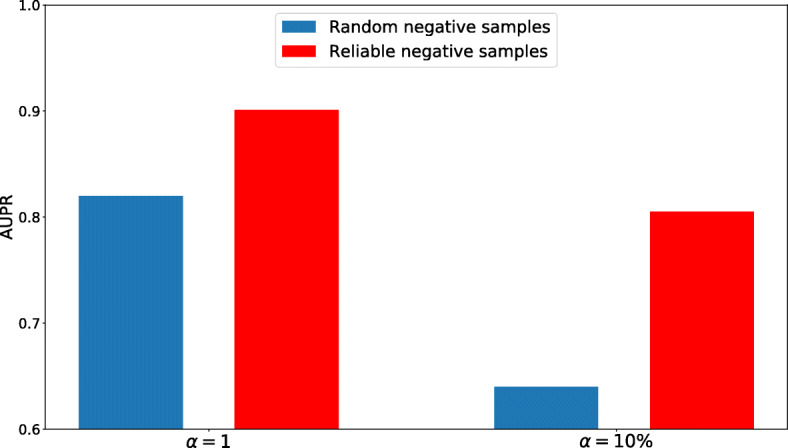
Comparison of the performance of our model with random negative samples and reliable negative samples in terms of AUPR

To investigate how negative sampling technique affects the performance of our model, we compared the performance of our model with negative sampling technique mentioned in Subsection “Pre-processing” and random sampling of unknown interactions. The 10-fold cross validation results in terms of AUROC and AUPR are provided in Figs. 8 and 9, respectively. As expected, the performance when reliable negatives are used for training is higher than randomly selected negative samples. The importance of using reliable negative samples can be even more pronounced where positive to negative ratio *α* is low (i.e. 10%).

We additionally tested our method on four datasets introduced in [46] (so-called Yamanishi dataset). These datasets correspond to four different target protein types, namely nuclear receptors (NR), G protein-coupled receptors (GPCR), ion channels (IC) and enzymes (E). Dataset specification is provided in Additional file 1: Table S1. Results in Additional file 1: Figure S1 show our approach achieved consistent results in Yamanishi dataset. For NR dataset, the performance is relatively lower than other categories. We surmise this happens due to lack of enough training data.

## Discussion

Although our methodology is not fully end-to-end learning, it eliminates the use of hand-crafted features and lets neural network learns features based DTI network. An important step in our methodology is to capture the network topology surrounding drug-target link by enclosing sub-graphs. All first-order heuristics such as common neighbors can be calculated from the 1-hop enclosing sub-graphs. However, researchers have shown that high-order heuristics such as Katz perform much better than first and second-order methods [47]. This is reflected in our comparisons shown in Fig. 5. To effectively learn high-order features, one may think that a very large hop number *h* is always needed. However, this leads to very large enclosing sub-graph which dramatically increases the computational complexities. Moreover, Zhang et al. showed that we do not necessarily need a very large *h* to learn high-order graph structure [24]. The authors reported that features can be learnt using even small *h*-hop sub-graphs. This can indirectly be observed in Fig. 7 which shows the performance of our model quickly ramps up when number of nodes (*K* which is proportional to *h*) in sub-graph increases.

Our methodology, similar to other graph/node labeling techniques, relies on preserving two key attributes, i.e. structural role topological directionality [24, 25]. Specifically in our approach, Pallete-WL algorithm (Subsection “Sub-graph pattern encoding”) achieves this preservation by labeling structural differences hence providing additional information to facilitate training process.

Although our neural network approach has advantage over methods that use hand-crafted features by learning from network topology information, it has some limitations. Firstly, our method trains a fully-connected neural network on flattened upper triangular of adjacency matrices (see Fig. 1 and its explanation) Since fully-connected neural networks only accept fixed size feature vectors as input, sub-graphs with different sizes need to be truncated. Consequently, our method may not consistently learn from the full *h*-hop neighborhood of each link and may miss some structural information which may limit our model’s performance. Secondly, due to the limitation of adjacency matrix representations, our approach cannot learn from explicit features [24].

Very recently, other type of relations such as drug-drug and protein-protein interactions, drug-disease and drug-side-effect associations have been considered for DTI prediction by researchers [28, 32, 48]. In future, we intend to incorporate these associations within our methodology.

We acknowledge that ultimate validation of drug-target prediction is to show how the prediction method can re-discover some FDA-approved drugs. We can certainly generate the top (highest prediction scores) of drug-target pairs for further inspection. However, full-fledge validation requires a much more comprehensive study of the FDA-approved drugs that is beyond the scope of this work.

## Conclusion

We have proposed a DTI prediction methodology using drug-target network, drug structural similarities and protein sequence similarities. We modeled this problem as link prediction in a semi-bipartite graph and used deep learning as a learning tool. One advantage of our model is that, it captures more useful relational information and automatically learns topological features from DTI network. Additionally, it uses neural networks to learn complex topological features which heuristics cannot express. Through comprehensive experimentation, we have shown that our model achieves better performance compared to other methods reported in literature.

## Supplementary information

**Additional file 1** Supplementary table and figure.

## Data Availability

The datasets used in this project can be found in: https://github.com/HafezEM/DTI
