# Supplementary material for: Drug-target interaction prediction using semi-bipartite graph model and deep learning
Source: BMC Bioinformatics. 2020 Jul 6;21(Suppl 4):248. doi: 10.1186/s12859-020-3518-6 (PMC7336396; doi:10.1186/s12859-020-3518-6)
Supplement: Supplementary file 1 — Additional file 1 Supplementary table and figure. [file 12859_2020_3518_MOESM1_ESM.pdf]

Table S1: Dataset specification. The number of drugs, targets, interaction among them and the drug-target interaction ratio.

Dataset	IC	Enzyme	GPCR	NR
Drugs (D)	204	445	95	54
Targets (T)	210	664	223	26
Known Interactions	1476	2926	635	90
DTI Ratio	3.4%	1%	3%	6.4 %

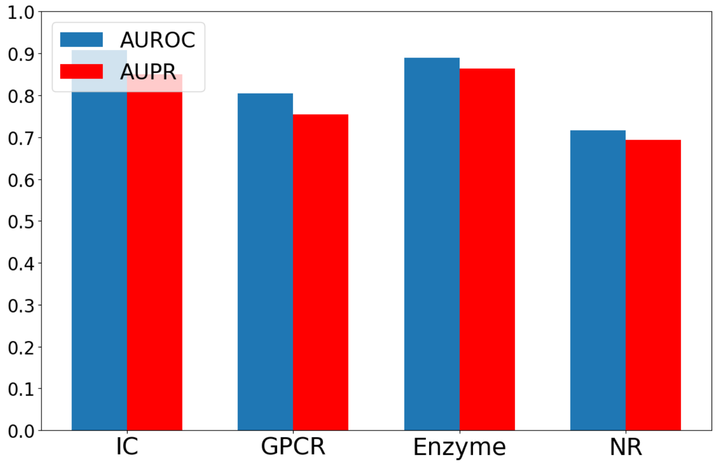

Figure S1: Performance on Yamanishi dataset in terms of AUROC and AUPR scores.
